# Patients with Liver Cirrhosis as Frequent Attenders of Emergency Departments

**DOI:** 10.1155/2020/8289275

**Published:** 2020-02-11

**Authors:** Chen-Ju Chuang, Yi-Fang Wu, Kai-Hsiang Wu, Yi-Chuan Chen

**Affiliations:** ^1^Department of Emergency Medicine, Chang Gung Memorial Hospital, Chiayi, No. 6, W. Sec., Jiapu Rd., Puzih City, Chiayi County 613, Taiwan; ^2^Chang Gung University College of Medicine, No. 5, Fusing St., Gueishan Township, Taoyuan County 333, Taiwan

## Abstract

**Objectives:**

Frequent attendance for nonemergency problems to emergency departments (EDs) contributes to ED overcrowding, resulting in medical care delays, increased medical errors, and social and economic burdens. Most studies regarding frequent attenders of EDs examine general patients without classifying certain subgroups. This study aimed to investigate patients with liver cirrhosis who present repeatedly to the ED.

**Methods:**

This was a retrospective, observational cohort study of adult patients with a history of liver cirrhosis presenting to the ED from January 2011 to December 2015. We included patients with cirrhosis whose first ED visit occurred during the study period. We went far back for 20 years and excluded patients with any ED visits (including both cirrhosis and noncirrhosis-related ones) before the study period. We categorized frequent attenders as patients with more than 4 ED visits within 12 months after the first ED visit; infrequent attenders were those who did not meet this criterion.

**Results:**

A total of 3513 patients with cirrhosis were included in this retrospective cohort study. Compared with the infrequent attenders, frequent attenders had a higher rate of presentations due to hepatic encephalopathy (15.2% vs 13.7%, *P* < 0.001) and ascites (10% vs 4%, *P* < 0.001) and ascites (10% vs 4%, *P* < 0.001) and ascites (10% vs 4%,

**Conclusions:**

Hepatic encephalopathy and ascites account for more ED visits in frequent than in infrequent attenders. Our findings provide information for those planning outpatient support for patients with cirrhosis. Further research is warranted.

## 1. Introduction

Overcrowding of emergency departments (EDs) has become a serious problem due to patient demand exceeding the department capacity. A cross-sectional survey of 243 Canadian ED directors found that approximately 62% of Canadian EDs were at or over capacity in 2005. [1] Between 1996 and 2013, ED attendance in Singapore nearly doubled. [2, 3] A survey conducted by the American Hospital Association reported that the percentage of large hospital EDs that are consistently operating at or above capacity has reached 90%. [4] The same problem has appeared in Taiwan in recent years. [5] This situation not only consumes valuable medical resources but also reduces medical quality due to overloaded health care providers. Evidence from a systematic review has indicated that frequent attenders account for 4.5%–8% of all ED patients and contribute to 21%–28% of all visits. [6].

A unique subset of frequent ED attenders is patients with cirrhosis of the liver. According to a study conducted in 2010, liver cirrhosis ranked as the 23rd cause of global disease burden with 31 million (1.2%) cases worldwide. [7] The prevalence of cirrhosis in the United States (US) was approximately 0.27%, corresponding to 633,323 adults; cirrhosis mortality was 26.4% per 2-year interval between 1999 and 2010. [8] Many visits that feel necessary to frequent attenders appear unnecessary to the health care professionals that attend them. [9] However, few studies describe the patients who frequently use the ED for liver cirrhosis-related complications. The purpose of this study was to describe this population and their reasons for ED attendance.

## 2. Materials and Methods

### 2.1. Study Population

This was a retrospective, observational cohort study of adult patients with history of liver cirrhosis presenting to the ED from January 2011 to December 2015. We included patients with cirrhosis whose first ED visit occurred during the study period. We went far back for 20 years and excluded patients with any ED visits (including both cirrhosis and noncirrhosis related ones) before the study period. This present study used a database of electronic medical records from the Chang Gung Research Database. Our study collected data from six hospitals with various levels of medical care, including two medical centers, three regional hospitals, and one local hospital.

The confidentiality of the medical records was ensured by anonymization and deidentification. The Institutional Review Board of Chang Gung Medical Foundation approved the study protocol (IRB No. 201600990B0C102). Informed consent was waived due to the anonymized nature of data used in this study.

### 2.2. Definitions

The definition of a frequent attender was adapted from other previous studies [2, 10].

Patients with >4 ED visits within 12 months after the first ED visit were categorized as frequent attenders. Patients who had been followed up for >12 months were categorized as frequent attenders if they had >4 ED visits in any one 12-month period. For example, if one came twice in the first year and 6 times in the second, that patient would be categorized as a frequent attender. Infrequent attenders were those who did not meet this criterion.

Most definitions of our study were based on a recent study discussing the association between bacteremia and gastrointestinal (GI) bleeding in patients with liver cirrhosis from Taiwan [11] Liver cirrhosis was diagnosed according to the findings of abdominal sonography with concomitantly laboratory evidences of hepatic dysfunction or clinical features of portal hypertension. The severity of liver cirrhosis was categorized on the basis of Child–Pugh (CP) classification [11].

An infection-related complication was defined as any finding of infection, such as bacteremia, spontaneous bacterial peritonitis, pneumonia, or urinary tract infection (UTI). The definition of bacteremia was any positive detection of bacteria via the blood cultures during hospital stay. The definition of spontaneous bacterial peritonitis (SBP) was an ascites fluid containing polymorphonuclear cell count >250 cells/mm^3^. The diagnosis of pneumonia was based on the clinical symptoms and signs, such as fever, productive cough, pleuritic chest pain, and rales, accompanied with abnormal chest X-ray findings. UTI was diagnosed as a positive urine culture with a bacterial colony count >105 colony-forming units/mL [11].

The diagnosis of GI bleed was based on the initial chief complaints including any one of the following: vomiting red or black blood, bloody stool, or black stool passage. A spectrum of neurological impairments or neuropsychiatric anomalies observed during the hospital stay was considered as Hepatic encephalopathy (HE) [11]. The diagnosis of ascites was based on the chief complaints of the fluid accumulation of peritoneal cavity and requests for control. The comorbidities were based on each patient's medical history as documented on admission or outpatient department records. We followed the methods of Shih et al. 2018 [11]. To avoid unreliable or biased abstractions that may result from undefined chart review procedures, we followed a number of techniques recommended for retrospective chart review [12, 13].

The primary data evaluation of our study was the survival during the study period. Enrolled patients were followed throughout the study period or until death occurred.

### 2.3. Statistical Analysis

The statistical analysis was performed using MedCalc Statistical Software version 17.0.4 (MedCalc Software bvba, Ostend, Belgium; https://www.medcalc.org; 2017). The enrolled patients were divided into frequent and infrequent groups. The normally distributed data were presented as mean with standard deviation, and the data with skewed distribution were expressed as median and interquartile range. The Mann–Whitney *U* and chi-squared tests were used to test the differences between the two groups. The difference was considered significant if the *P* value was <0.05. The Kaplan–Meier method was used to analyze the survival of patients with cirrhosis who did or did not make frequent ED visits during the study period. A log-rank test was performed to examine the differences in survival. Logistic regression analysis was used for factors associated with frequent attenders.

## 3. Results

The records of 3513 patients with cirrhosis were evaluated. Of the 3513 patients, 2429 (69.1%) were defined as the infrequent group and 1084 (30.9%) as the frequent group.  Table 1 shows the characteristics for both the cirrhosis-related infrequent attenders and the frequent attenders.

Table 2 shows attendance characteristics for cirrhosis-related infrequent attenders and frequent attenders. The total number of ED visits from infrequent attenders was 9059 (33.5%), whereas the number of frequent attenders totaled 18,882 (66.5%). The reasons for the visits were classified into four groups: infection related, GI bleeding, HE, and ascites. Compared with the infrequent attenders, the frequent attenders had a higher proportion of visits due to HE (15.2% vs 13.7; *P* < 0.001) and ascites (10% vs 4%; *P* < 0.001). Additional patient characteristics and visit characteristics are depicted in each table.

**Figure 1:**
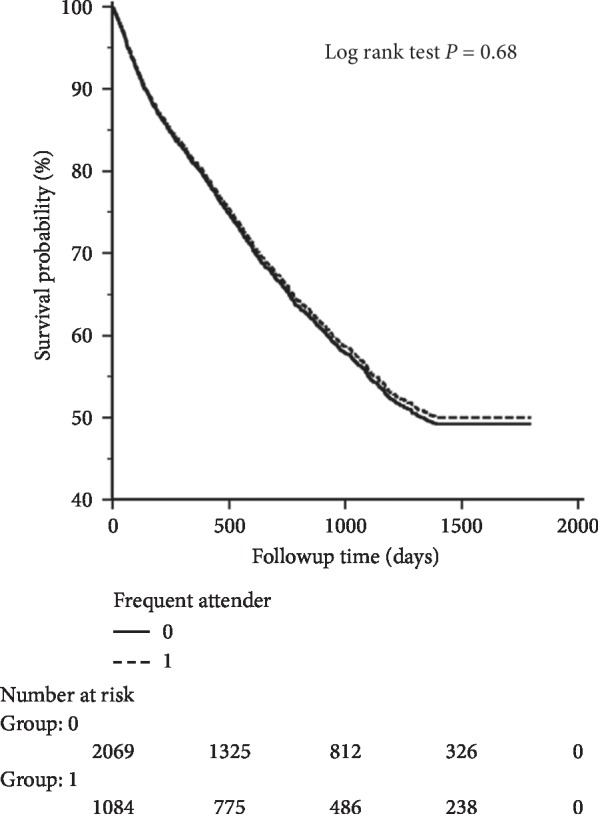
Kaplan–Meier (KM) curve for study-period survival.

Table 3 shows the results of the Kaplan–Meir survival analysis. Frequent attenders were not associated with increased mortality during the study period (hazard ratio [HR] 1.02; *P*=0.68) (Figure 1).

The results of logistic regression for factors associated with frequent attenders are displayed in Supplement Table 1.

## 4. Discussion

Since 2011, liver cirrhosis has been included the first 10 causes of death in Taiwan and accounted for a mortality rate of 19.3 per 100,000 population in 2017 [14]. In the US, cirrhosis is the twelfth overall cause of death and the second most common digestive disease cause of death. [15] With a mortality rate of 25.7 deaths per 100,000 people, cirrhosis and its complications account for approximately 40,000 deaths in the US annually, similar to diabetes and slightly more than kidney diseases. [16] In the United Kingdom, mortality from cirrhosis has increased from 6 per 100,000 population in 1993 to 12.7 per 100,000 population in 2000. [17] A huge increase in the burden of liver disease is expected over the years, with an inevitable increase in cirrhosis complications. [18] In our study, we identified 1084 patients as frequent ED attenders from 2011 to 2015. The most frequent attender had a total of 398 ED visits during the study period. This increases the burden on the medical staff, leading to poor quality of treatment, may increase the conflicts between patients and medical care providers. Age difference between frequent and infrequent attenders was noted. The possible reason for younger age in frequent attender group might be that their desire for convenient care that fits around work schedules and other obligations. In our study, frequent attenders had lower rate of ED visits in the daytime and weekday. One report documented that younger patients were more likely to visit the ED because of their need to seek care outside regular business hour. [19] One recent study in Germany revealed that the onset of frequent attendance was negatively associated with age (odds ratio (OR): 0.91, 95% CI: 0.87–0.95) [20]. We examined several variables between the frequent and infrequent attender groups. First, the rate of major diseases was higher in the frequent attender than in the infrequent attender group. These diseases include heart failure, cerebrovascular disease, chronic obstructive pulmonary disease, malignancy other than hepatocellular carcinoma, and alcoholic liver cirrhosis. This result is consistent with other research showing high comorbidity of cirrhosis with the highest prevalence for chronic obstructive lung disease (7.3%), cancer (6.7%), and heart failure (5.2%) [7]. Liver cirrhosis severity was higher in the frequent attender group. The CP score and Model for End-Stage Liver Disease score were both higher. There were also more total ED visits, total admissions, admissions through the ED, and total lengths of hospital stay for all admissions for the frequent attender group. These statistical data were reasonable because more comorbidities could result in greater complications, increasing the admission rate and extending the treatment course, consistent with the previous reports [4].

However, more nonurgent or nonemergent symptoms and signs accounted for the main causes of ED visits in the frequent attender group when the characteristics of ED visits were analyzed. In the frequent attender group, we found that the computed tomography examination times, laboratory testing times, and total length of hours spent at the ED were less than in the infrequent attender group; triage level, general ward admission, and intensive care unit admission rates were also lower in the frequent attender group. These data indicated that most of the ED visits by frequent attenders were for less acute illnesses, but an HE or workup for SBP was still needed. We would need more detail on their ED course before we could suggest that finding an alternative venue for these patients would result in better outcomes for the system or the patients. We performed a further analysis of cirrhosis-related visit characteristics for infrequent and frequent attenders. The two major causes for which infrequent attenders sought help were GI bleeding and infection-related complications, whereas for the frequent attenders, it was HE and ascites-related complications, such as abdominal distention or abdominal pain.

GI bleeding was a common complication for patients with cirrhosis related to portal hypertension, coagulopathy, and thrombocytopenia. Variceal bleeding accounted for 59% of upper GI bleeding in patients with cirrhosis, followed by peptic ulcer disease in 16% of cases [21]. In-hospital mortality rates for any type of GI bleeding in patients with cirrhosis are essentially double those of patients without cirrhosis [22]. Due to its high mortality rate, upper GI bleeding in liver cirrhosis is indeed a serious issue. The other major risk leading to death was infection. In clinical practice, bacteremia in patients with cirrhosis is very common, causing fever, abdominal pain, dyspnea, shock, disturbed consciousness, and even death. According to other studies, sepsis is the leading cause of hospitalization and death in intensive care units [23]. Common bacterial infections in patients with cirrhosis include SBP, UTI, pneumonia, bacteremia, and soft-tissue infections, which directly cause 30%–50% of deaths in patients with cirrhosis [24].

In contrast, in our cohort of frequent attenders, the most common causes of ED visits were HE and ascites. HE was the most common, possibly preventable, cause for readmission [25]. To prevent repeated hospitalizations and enhance better understanding of the management of HE in specific patients, close liaisons should be made with the patient's family, the general practitioner, and other primary healthcare providers [26].

Ascites is the most common reason for hospitalization of patients with cirrhosis in the US [25]. Outpatient treatment can initially be attempted for patients with uncomplicated ascites, especially those without GI bleeding, HE, bacterial infections, hepatocellular carcinoma, hypotension, or azotemia [18]. Ascites was the second possibly preventable cause of readmission [25]. Reduced readmission rates for patients with cirrhosis and ascites could be achieved with a rapid return to outpatient or clinical services, frequent adjustment of dosage of diuretics, and prevention of dehydration [18]. Another important finding in this study was that frequent attendance was not associated with increased mortality during the study period (HR 1.02; *P*=0.68). Although their Child–Pugh classification was less severe, the infrequent attenders were more likely to have immediately life-threatening reasons for visits. We deduced that they are a subgroup that only comes if very ill. The frequent attenders come more often, experience less dramatic acute issues (GI bleeds/infection), and, in spite of their more severe cirrhosis, die at the same rate as do infrequent attenders. This suggests that perhaps the frequent ED visits result in better control and more attentive attitudes toward health. This finding implies that because frequent attenders have fewer admissions and are more likely to present with conditions that could conceivably be delivered in a more appropriate venue than the ED, an easily accessible outpatient service may offer a venue that patients with cirrhosis may use in place of the ED. This would potentially be more convenient for patients and save costs for the system.

This study is subject to several limitations. First is the retrospective nature of the study. Second, our database does not have data on family supports and patient's comprehensions to the disease course and complications. Patients' data outside our institutions is unavailable. Third is the degree of influence of confounders, including comorbidities, medications, and facilities at different EDs. In addition, patients admitted to hospital through outpatient services were not enrolled in our study. These limitations could have caused potential bias.

## 5. Conclusions

Our study identified that this frequent attender population has a higher rate of medical comorbidities and more visits resulting from HE and ascites, compared with infrequent attenders. Further studies focusing on the causes of visits and multisystem care plan for cirrhotic patients are suggested.

## Figures and Tables

**Table 1:** Characteristics for cirrhosis-related infrequent attenders and frequent attenders.

Characteristics	Infrequent attender group		Frequent attender group		*P* value
	*N* = 2429	69.1%	*N* = 1084	30.9%	
Male	1712	70.5	778	71.8	0.44
Age (IQR)	60 (50–70)		58 (47–69)		<0.001
Ischemic heart disease	199	8.2	173	16.0	<0.001
Heart failure	112	4.6	118	10.9	<0.001
Cerebrovascular accidents	74	3.0	59	5.4	<0.001
Peripheral vascular disease	48	2.0	33	3.0	0.051
COPD	1088	44.8	698	64.4	<0.001
Chronic renal disease	411	16.9	324	29.9	<0.001
Malignancy other than HCC	300	12.4	237	21.9	<0.001
HCC	1045	43.0	433	39.9	0.09
Alcoholic liver cirrhosis	571	23.5	378	34.9	<0.001
Liver cirrhosis severity					
Child A	796	32.8	275	25.4	<0.001
Child B	1078	44.4	478	44.1	0.88
Child C	555	22.8	331	30.5	<0.001
MELD (IQR)	10.0 (4.2–15.8)		11.1 (4.0–17.8)		0.036
Total ED visits (IQR)	3 (2–5)		13 (9–20)		<0.001
Total admission times (IQR)	3 (2–5)		8 (5–12)		<0.001
Admission through ED (times) (IQR)	2 (1–3)		6 (4–9)		<0.001
Total hospital length of stay for all admissions (IQR)	30 (14–56)		74 (41–123)		<0.001

Child A, B, and C: Child–Pugh classification A, B, and C (child A: good hepatic function; child B: intermediate hepatic function; child C: poor hepatic function); IQR: interquartile range; COPD: chronic obstructive pulmonary disease; HCC: hepatocellular carcinoma; MELD: Model for End-Stage Liver Disease; ED: emergency department.

**Table 2:** Visit characteristics for cirrhosis-related infrequent attenders and frequent attenders.

Characteristics	Infrequent attender group		Frequent attender group		*P* value
	*N* = 9059	33.5%	*N* = 18882	66.5%	
Daytime	5060	53.2	9594	50.8	<0.001
Weekend	2487	26.2	5153	27.3	0.042
Triage 1 or 2	3403	35.8	4431	23.5	<0.001
EMS transport	1716	18.1	2479	13.1	<0.001

*Causes of encounter*					
Infection-related	1257	13.2	2239	11.9	0.001
GI bleeding	3880	45.8	3245	17.2	<0.001
Hepatic encephalopathy	1307	13.7	2871	15.2	0.001
Ascites	386	4.0	1888	10.0	<0.001

*ED management*					
CT examination	1228	12.9	1932	10.2	<0.001
Laboratory testing	8558	90.0	14066	47.5	<0.001
Length of stay (hours) (IQR)	16.7 (3.4–48.9)		5.2 (1.6–26.6)		<0.001

*Admission status*					
Hospital admission	5027	52.9	7030	37.2	<0.001
ICU admission	94	1.0	72	0.4	<0.001

EMS: emergency medical services; GI: gastrointestinal; ED: emergency department; CT: computed tomography; IQR: interquartile range; ICU: intensive care unit.

**Table 3:** Survival during study period.

Variable	Hazard ratio	*P* value
Frequent attender	1.02	0.68

## Data Availability

The datasets used and analyzed during the current study are available from the corresponding author on reasonable request.

## Supplementary Materials

Supplementary Materials Supplement Table 1: logistic regression analysis of factors associated with presenting as frequent attenders.
