# Supplementary material for: Patients with Liver Cirrhosis as Frequent Attenders of Emergency Departments
Source: Emerg Med Int. 2020 Feb 11;2020:8289275. doi: 10.1155/2020/8289275 (PMC7036124; doi:10.1155/2020/8289275)
Supplement: Supplementary Materials — Supplement Table 1: logistic regression analysis of factors associated with presenting as frequent attenders. [file 8289275.f1.pdf]

Supplement Table 1

Logistic regression analysis of factors associated with presenting as frequent attenders

**Variables**	**Adjusted odds ratio**	**95% CI**	**p value**
**Age**	0.980	0.974-0.987	< 0.001
**Ischemic heart disease**	1.687	1.315-2.163	< 0.001
**Heart failure**	1.883	1.381-2.569	< 0.001
**COPD**	2.053	1.745-2.416	< 0.001
**Chronic renal disease**	1.843	1.526-2.226	< 0.001
**Malignancy other than HCC**	1.967	1.604-2.412	< 0.001
**Alcoholic liver cirrhosis**	1.394	1.155-1.682	< 0.001
**Liver cirrhosis Child A**	0.817	0.676-0.987	0.036
**Liver cirrhosis Child C**	1.249	1.036-1.507	0.020

COPD: chronic obstructive pulmonary disease; HCC: hepatocellular carcinoma; Child A, B, C: Child-Pugh classification A, B, C (Child A: good hepatic function; Child B: intermediate hepatic function; Child C: poor hepatic function)

Hosmer-Lemeshow test for the model: P = 0.160
